# Supplementary material for: Analysis of PPARγ Signaling Activity in Psoriasis
Source: Int J Mol Sci. 2021 Aug 10;22(16):8603. doi: 10.3390/ijms22168603 (PMC8395241; doi:10.3390/ijms22168603)
Supplement: Supplementary file 1 [file ijms-22-08603-s001.zip › Supplemental materials_Analysis of PPARg signaling activity in psoriasis/Pathway models/Models images and html files/PPARG expressed in cells assotiated with psoriasis/225564.html]

leukocyte


# CellType leukocyte

|  |  |
| --- | --- |
| URN | urn:agi-ncimcelltype:C1550647 |
| Total Entities | 69 |
| Connectivity | 9950 |
| Name | leukocyte |

---

|  |  |
| --- | --- |
| ChildConcepts | leukocyte function |
|  | leukocyte interaction |
|  | leukocyte aggregation |
|  | leukocyte structure |
|  | leukocyte migration |
|  | immune cell homeostasis |
|  | leucocyte differentiation |
|  | immune cell chemotaxis |
|  | leukocyte cell adhesion |
|  | leukocyte proliferation |
|  | leukocyte tethering / rolling |
|  | leukocyte apoptosis |
|  | mononuclear leukocyte |
|  | granulocyte |
|  | mononuclear cell |
|  | leukocyte infiltration |
|  | leukocyte transendothelial migration |
|  | leukocyte population |
|  | leukocyte homing |
|  | leukocyte diapedesis |
|  | leukocyte formation |
|  | leukocyte transdifferentiation |
|  | leukocyte destruction |
|  | leukocyte polarity |
|  | leukocyte phagocytosis |
|  | leukocyte sequestration |
|  | leukocyte contact |
|  | leukocyte proliferative response |
|  | leukocyte localization |
|  | leukocyte phenotype |
|  | leukocyte growth |
|  | leukocyte survival |
|  | leukocyte transepithelial migration |
|  | leukocyte fate |
|  | leukocyte distribution |
|  | leukocyte motion |
|  | leukocyte recognition |
|  | leukocyte development |
|  | leukocyte renewal |
|  | leukocyte communication |
|  | leukocyte invasion |
|  | leukocyte death |
|  | leukocyte mediated cytotoxicity |
|  | leukocyte spreading |
|  | leukocyte damage |
|  | leukocyte priming |
|  | leukocyte ratio |
|  | leukocyte agglutination |
|  | leukocyte viability |
|  | leukocyte lifespan |
|  | leukocyte regeneration |
|  | leukocyte cytotoxicity |
|  | leukocyte division |
|  | leukocyte swelling |
|  | leukocyte repertoire |
|  | leukocyte selection |
|  | leukocyte phagocyte activity |
|  | leukocyte compartmentalization |
|  | leukocyte mononuclear cell migration |
|  | leukocyte disruption |
|  | leukocyte size |
|  | leukocyte anchorage |
|  | leukocyte stress |
|  | leukocyte replicative senescence |
|  | leukocyte senescence |
|  | leukocyte autophagy |
|  | leukocyte volume |
|  | leukocyte granulocyte differentiation |
|  | leukocyte elongation |

---

|  |  |
| --- | --- |
| Pathway | LDLR -> Expression Targets in Lymphoid System and Blood |
|  | TGF-beta Receptors Family -> Expression Targets in Lymphoid System and Blood |
|  | EGFR-> Expression Targets in Lymphoid System and Blood |
|  | F2R/F2RL1 -> Expression Targets in Lymphoid System and Blood |
|  | CD Markers -> Expression Targets in Lymphoid System and Blood |
|  | TNF Receptors -> Expression Targets in Lymphoid System and Blood |
|  | Immunoglobulin Superfamily -> Expression Targets in Lymphoid System and Blood |
|  | Interleukin Receptors -> Expression Targets in Lymphoid System and Blood |
|  | C5AR1 -> Expression Targets in Lymphoid System and Blood |
|  | IgE Receptors -> Targets in Lymphoid System and Blood |
|  | Chemokine Receptor Family -> Expression Targets in Lymphoid System |
|  | PPARG expressed in cells assotiated with psoriasis |
|  | Neighbors of leukocyte |

---

|  |  |
| --- | --- |
| MedScan ID | 10000079 |

---

|  |  |
| --- | --- |
| Alias | peripheral blood leukocyte |
|  | peripheral leukocyte |
|  | peripheral leukocytes |
|  | leukocyte |
|  | white blood cells |
|  | leukocytes |
|  | white cells |
|  | peripheral blood leukocytes |
|  | white blood corpuscle |
|  | white blood corpuscles |
|  | leukocytic |
|  | white blood cell |
|  | white cell |

---

|  |  |
| --- | --- |
| NCIm ID | C1550647 |

---
